# Neuroprotective Activity of Thioctic Acid in Central Nervous System Lesions Consequent to Peripheral Nerve Injury

**DOI:** 10.1155/2013/985093

**Published:** 2013-12-29

**Authors:** Daniele Tomassoni, Francesco Amenta, Lorenzo Di Cesare Mannelli, Carla Ghelardini, Innocent E. Nwankwo, Alessandra Pacini, Seyed Khosrow Tayebati

**Affiliations:** ^1^School of Bioscience and Biotechnology, University of Camerino, Via Gentile III da Varano, 62032 Camerino, Italy; ^2^School of Medicinal and Health Products Sciences, University of Camerino, Via Madonna delle Carceri, 9, 62032 Camerino, Italy; ^3^Department of Clinical and Preclinical Pharmacology, University of Florence, Viale Pieraccini, 6, 50134 Florence, Italy; ^4^Department of Anatomy, Histology and Forensic Medicine, University of Florence, Largo Brambilla, 1, 50134 Florence, Italy

## Abstract

Peripheral neuropathies are heterogeneous disorders presenting often with hyperalgesia and allodynia. This study has assessed if chronic constriction injury (CCI) of sciatic nerve is accompanied by increased oxidative stress and central nervous system (CNS) changes and if these changes are sensitive to treatment with thioctic acid. Thioctic acid is a naturally occurring antioxidant existing in two optical isomers (+)- and (−)-thioctic acid and in the racemic form. It has been proposed for treating disorders associated with increased oxidative stress. Sciatic nerve CCI was made in spontaneously hypertensive rats (SHRs) and in normotensive reference cohorts. Rats were untreated or treated intraperitoneally for 14 days with (+/−)-, (+)-, or (−)-thioctic acid. Oxidative stress, astrogliosis, myelin sheets status, and neuronal injury in motor and sensory cerebrocortical areas were assessed. Increase of oxidative stress markers, astrogliosis, and neuronal damage accompanied by a decreased expression of neurofilament were observed in SHR. This phenomenon was more pronounced after CCI. Thioctic acid countered astrogliosis and neuronal damage, (+)-thioctic acid being more active than (+/−)- or (−)-enantiomers. These findings suggest a neuroprotective activity of thioctic acid on CNS lesions consequent to CCI and that the compound may represent a therapeutic option for entrapment neuropathies.

## 1. Introduction

Lesions of the nervous system can induce dysfunctional pain signalling and altered sensory mechanisms identifying a heterogeneous category of diseases defined neuropathies. These pathologies are difficult to treat. In general, drugs available counter hyperalgesic symptomatology of neuropathy, but do not affect the course of these diseases. Neuroprotective and/or neurorestorative effects elicited by pharmacological treatments were reported only rarely [1].

Chronic constriction injury (CCI) is an animal model of peripheral neuropathy induced by the loose ligation of the sciatic nerve [2]. CCI mimics an entrapment mononeuropathy and is characterized by a painful syndrome with hyperalgesia. Painful symptomatology starts approximately from the 3rd day after nerve injury, reaches a plateau between 7 and 15 days, and then decreases [3]. In CCI, hyperalgesia is accompanied by the occurrence of apoptosis phenomena in the nerve starting from the second week after ligation [4].

Treatment of neuropathic pain, initiated or caused by central nervous system (CNS) primary lesions/dysfunctions or by peripheral nervous system (nerves outside the brain and spinal cord) damage is problematic because of severity, chronicity, and resistance to common analgesics [5]. Reactive oxygen species (ROS) have been implicated in the development of persistent pain states resulting from nerve injury or inflammatory phenomena [6–10]. Several studies have shown that antioxidants are effective in alleviating hyperalgesia in spinal nerve-ligated neuropathic rats [11, 12] and capsaicin-induced secondary mechanical hyperalgesia in rats and mice. Increasing evidence supports the view that ROS are a pathogenetic factor for the development and maintenance of persistent neuropathic pain [13].

Morphological changes of some brain areas including the prefrontal cortex were reported in patients experiencing chronic pain [14, 15]. Structural and functional reorganization of the medial prefrontal cortex was also found in rats affected by neuropathic pain [16]. Although these studies have suggested a correlation between neuropathic pain and cognitive dysfunction, there is still much to learn on how chronic pain may affect brain function and to identify cellular mechanisms underlying cognitive disturbances taking place in chronic pain conditions.

Thioctic acid is as a naturally occurring (biological) antioxidant and detoxifying agent proposed for treating diabetic neuropathy, for countering age-associated cardiovascular, cognitive, and neuromuscular deficits, and as a modulator of various inflammatory signaling pathways [17]. The pharmacology of thioctic acid and its role as biological antioxidant, neuroprotectant, and agent interfering in liver metabolism and disease were reviewed [18–21]. Due to the presence of an asymmetric carbon C3, thioctic acid exists in two enantiomers, namely, (+)- and (−)-thioctic acid. The latter enantiomer probably represents the active form of the compound. It is located intracellularly and elicits the biological effects of thioctic acid [17]. Thioctic acid is used worldwide as a nutraceutical or registered drug, and it is marketed mainly in the racemic (+/−)-thioctic acid form for stability reasons.

Rat strains with genetically inherited hypertension were developed since more than 50 years. The spontaneously hypertensive rat (SHR) is probably the model more widely investigated. It is characterized by arterial hypertension, increased oxidative stress, and overproduction of ROS [22]. Hence, SHR can be used as an animal model of oxidative stress and for investigating the activity of antioxidants.

The purpose of the present study was to assess if experimental compression of sciatic nerve, induced by loose ligation of it, is accompanied by an increased oxidative stress and by CNS changes. This study has also investigated the effect of treatment with enantiomers of thioctic acid on oxidative stress and on CNS damage induced by peripheral nerve injury, using western blotting and quantitative immunohistochemistry. Treatment with thioctic acid was compared to treatment with pregabalin, which is used as a standard pharmacological treatment of neuropathic pain [23].

## 2. Materials and Methods

### 2.1. Animals and Tissue Treatment

Twenty-week-old male SHR (*n* = 42) and age-matched WKY rats (*n* = 6) were used. Four rats were housed per cage (size 26 × 41 cm) and placed in the experimental room for acclimatization 24 h before testing. The animals were fed with standard laboratory diet and with tap water ad libitum and kept at 23 ± 1°C with a 12 h light/dark cycle, light at 7 a.m. Animal manipulations were carried out according to the National and European Community Guidelines for Animal Care (DL 116/92, application of the European Communities Council Directive 86/609/EEC) and ethical guidelines of the University of Florence. These guidelines are consistent with the Guide for the Care and Use of Laboratory Animals of the US National Institutes of Health (NIH Publication 85-23, revised 1996; University of Florence assurance number: A5278-01). All efforts were made to minimize animal suffering and to reduce the number of animals used.

### 2.2. Peripheral Mononeuropathy Rat Model

CCI is a model of peripheral neuropathy induced by the loose ligation of the sciatic nerve [2]. Briefly, rats were anaesthetized with 400 mg/kg chloral hydrate intraperitoneally (i.p.). Under aseptic conditions, the right common sciatic nerve was exposed at the level of the middle thigh by blunt dissection. Proximal to the nerve trifurcation, connective tissue surrounding it was carefully removed and four chromic cat gut ligatures (4–0, Ethicon, Norderstedt, Germany) were tied loosely around the nerve with about 1 mm spacing. After hemostasis was confirmed, incision was closed in layers. Animals were allowed to recover from surgery and then housed one per cage with free access to standard laboratory chow and water.

CCI operated animals were treated once a day for 14 days starting from the day of the operation with an i.p. injection of 250 *μ*mol/kg/day of (±)-thioctic acid (*n* = 6), 125 *μ*mol/kg/day of (±)-thioctic acid (*n* = 6), 125 *μ*mol/kg/day of (+)-thioctic acid lysine salt (*n* = 6), 125 *μ*mol/kg/day of (−)-thioctic acid (*n* = 6), and 300 *μ*mol/kg/day of pregabalin (*n* = 6). Sham-operated WKY (*n* = 6), Sham-operated SHR (*n* = 6) rats, and control CCI-operated SHR (*n* = 6) rats received the same amounts of vehicle.

Before killing animals were anaesthetised with pentobarbital sodium (50 mg/kg, i.p.), had 5 mL of blood collected by intracardiac withdrawal, and then were decapitated. In blood samples levels of thiobarbituric acid reactive substances (TBARS) and the activity of superoxide dismutase (SOD) were measured using commercial kits (Cayman Chemical Company, Cat. number 10009055 and Cat. number 706002, resp.). Plasma protein oxidation levels were also assessed by immunoblotting using a commercial kit (OxyBlot Protein Oxidation Kit, Millipore, Cat. number S7150).

The brain was removed from skull, washed, weighed, fixed in a Histochoice solution, and embedded in semi-synthetic paraffin. Serial coronal consecutive 8 *μ*m thick sections containing motor cortex (including zones 1 (layers I–IV) and 2 (layers V–VI) and corresponding white matter) (3.20 mm from Bregma, Plate 8) and sensory cortex (including zones 1 (layers I–IV) and 2 (layers V–VI) and corresponding white matter) (−0.30 mm from Bregma, Plate 19) [24] were stained with Nissl's method (cresyl violet 1.5%) for morphometric analysis and with hematoxylin and eosin for assessing the occurrence of relevant microanatomical changes. Serial consecutive coronal 12 *μ*m thick sections of the same area were processed for immunohistochemistry as detailed below.

### 2.3. Immunohistochemistry

Paraffin embedded coronal sections of the brain (12 *µ*m thick) were processed for the immunohistochemical detection of glial fibrillary acidic protein (GFAP) as a marker of astroglial reaction and myelin basic protein (MBP) as a marker of myelinated fibers. For investigating cerebrocortical neuronal components status, 200 kDa neurofilament (NFP) was used as axonal marker. 8-Oxo-2′-deoxyguanosine (8-oxo-dG) immunohistochemistry was used to analyse the DNA oxidative status.

The 1st, 7th, 13th, 19th, and 25th consecutive sections were processed for GFAP immunohistochemistry using a mouse serum against GFAP (Chemicon, Millipore, Cat. number 3402) diluted 1 : 500 with 0.3% PBS-Triton X 100. The 2nd, 8th, 14th, 20th, and 26th consecutive sections were processed for MBP immunohistochemistry by exposing them to a mouse monoclonal antibody raised against MBP (Chemicon, Millipore, Cat. number 5262) diluted 1 : 500. The 3rd, 9th, 15th, 21th, and 28th consecutive sections were processed for NFP immunohistochemistry by exposing them to a mouse monoclonal antibody raised against neurofilament 200 kDa (clone RT97, Chemicon, Millipore, Cat. number 5262) diluted 1 : 500. The 4th, 10th, 16th, 22th, and 28th were used as control sections and exposed to a non-immune sera instead of the primary antibody. The 5th, 11th, 17th, 23rd, and 29th were processed for 8-oxo-dG (clone 2E2, Trevigen, Cat. number 4354-MC-050) diluted 1 : 250 in PBS containing 0.1% of BSA. In accordance with the company protocol, the sections were pretreated at 37°C with 5 *μ*g/mL Proteinase K in PBS for 30 min and 100 *μ*g/mL RNase A in 15 mM sodium citrate buffer containing 150 mM NaCl for 60 min. The 6th slide of each consecutive series was stained with Nissl's method for assessing morphological details. For immunohistochemistry sections were exposed overnight in a moist chamber at 4°C to primary antibodies and then for 30 min at 25°C to corresponding secondary biotinylated antibodies (goat anti-mouse IgGs) diluted to 1 : 200. The product of immune reaction was revealed using 3,3′-diaminobenzidine as a chromogen.

### 2.4. Image Analysis

Nissl's stained sections were viewed under a light microscope at a final magnification of ×160. Via a TV connection, images were transferred from the microscope to the screen of an IAS 2000 image analyzer and used as a microanatomical reference for quantitative immunohistochemistry. The area of astrocytes, considered as cells displaying a dark-brown GFAP immunoreactivity, was assessed using on overlap function of the IAS 2000 image analyzer. Morphometric data were then analyzed according to the protocol described in an earlier paper of our group [25]. The density of immunoreaction area occupied by NFP or MBP was measured by image analysis in motor and sensory cortices by the image analysis protocol detailed elsewhere [26]. The intensity of NFP and MBP immunostaining developed in cerebrocortical axons was assessed microdensitometrically by calibrating the image analyzer taking “zero” as the background developed in sections incubated with a nonimmune serum and “250” as the conventional value of maximum intensity of staining.

The 8-oxo-dG immunostaining, developed in the nuclei of neurons of motor and sensory cortices, was also analyzed microdensitometrically.

### 2.5. Data Analysis

Means of different parameters investigated were calculated from single animal data, and group means ± SEM. were then derived from single animal values. The significance of differences between means was analyzed by analysis of variance (ANOVA) followed by the Newman-Keuls multiple range test.

## 3. Results

Body weight values were similar in normotensive WKY or SHR either control or treated with different formulations of thioctic acid or pregabalin. Brain weight values were lower in SHR either controls or treated compared to normotensive WKY rats (data not shown).

Systolic blood pressure values were higher in SHR rats compared to the normotensive WKY rats (Table 1). Ligation of the sciatic nerve and treatment with different enantiomers/dosages of thioctic acid or with pregabalin did not affect significantly blood pressure values in SHR (Table 1).

### 3.1. Plasma Analysis

**Figure 1:**
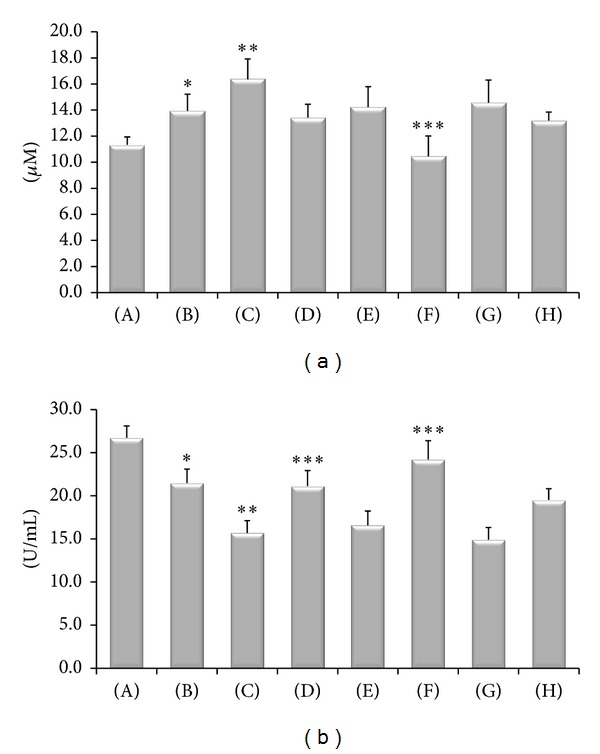
TBARS levels in plasma, expressed as *μ*M of malondialdehyde (a), and activity of SOD in plasma, expressed as U/mL (b). (A) Control Sham-operated WKY rats; (B) control Sham-operated SHRs; (C) control CCI SHRs; (D) CCI SHRs treated with (+/−)-thioctic acid 250 *μ*mol/kg/day; (E) CCI SHRs treated with (+/−)-thioctic acid 125 *μ*mol/kg/day; (F) CCI SHRs treated with (+)-thioctic acid 125 *μ*mol/kg/day; (G) CCI SHRs treated with (−)-thioctic acid 125 *μ*mol/kg/day; (H) CCI SHRs treated with pregabalin 300 *μ*mol/kg/day. *P < 0.05 versus WKY control rats; **P < 0.05 versus SHR Sham-operated control rats; ***P < 0.05 versus control CCI SHR.

**Figure 2:**
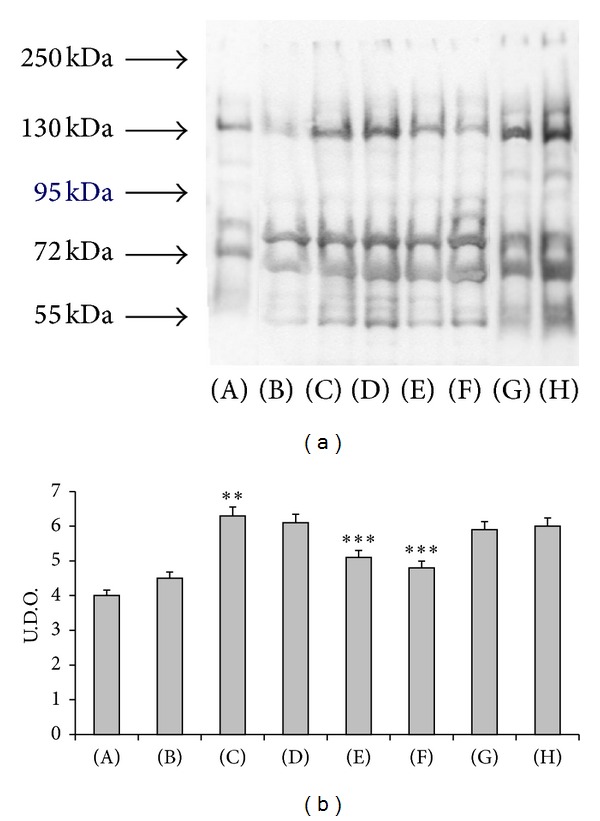
Western blot analysis of plasma oxidized protein (a) and densitometric analysis of these bands (b). The system revealed the oxidized plasmatic proteins with a range of molecular weight between 250 KDa and 55 KDa. (A) Control Sham-operated WKY rats, (B) control Sham-operated SHRs, (C) control CCI SHRs, (D) CCI SHRs treated with (+/−)-thioctic acid 125 *μ*mol/kg/day, (E) CCI SHRs treated with (+/−)-thioctic acid 250 *μ*mol/kg/day, (F) CCI SHRs treated with (+)-thioctic acid 125 *μ*mol/kg/day, (G) CCI SHRs treated with (−)-thioctic acid 125 *μ*mol/kg/day, and (H) CCI SHRs treated with pregabalin 300 *μ*mol/kg/day (H). Data of densitometric analysis were expressed as arbitrary units. **P < 0.05 versus SHR Sham-operated control rats; ***P < 0.05 versus control CCI SHR.

In SHR an increased oxidative stress characterized by a significant rise of plasma levels of TBARS (Figure 1(a)) with SOD decrease (Figure 1(b)) and increase of the protein oxidative status (Figure 2) was observed compared to WKY rats. Treatment with (+)-thioctic acid (125 *μ*mol/kg/day) significantly decreased TBARS (Figure 1(a)). The two different doses of (+/−)-thioctic acid had only a slight effect on TBARS (Figure 1(a)). SOD activity was also decreased in CCI-operated rats (Figure 1(b)). Treatment with higher dose of (+/−)-thioctic acid (250 *μ*mol/kg/day) and (+)-thioctic acid (125 *μ*mol/kg/day) significantly increased SOD activity (Figure 1(b)), whereas the lower doses of (+/−)-thioctic acid (125 *μ*mol/kg/day) and (−)-thioctic acid (125 *μ*mol/kg/day) were ineffective (Figure 1(b)). Immunoblotting analysis of protein oxidative status revealed an increase of oxidized protein levels in CCI-operated SHR (Figure 2). Treatment with (+/−)-thioctic acid at (250 *μ*mol/kg/day) and (+)-thioctic acid (125 *μ*mol/kg/day) countered a similar extent oxidative modification of plasma protein, whereas (−)-thioctic acid (125 *μ*mol/kg/day) or pregabalin was ineffective (Figure 2).

### 3.2. Motor and Sensory Cortex Immunohistochemistry

**Figure 3:**
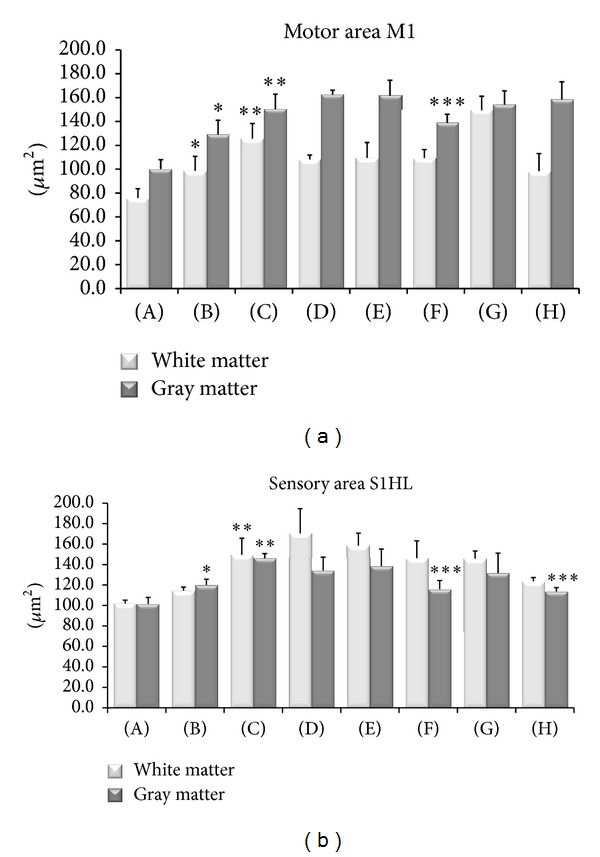
Size of GFAP-immunoreactive astrocytes in the cerebral cortex primary motor and sensory areas. Mean of immunoreactions areas of astrocytes is expressed in *μ*m^2^. Data are the mean ± SE. (A) Control Sham-operated WKY rats, (B) control Sham-operated SHRs, (C) control CCI SHRs, (D) CCI SHRs treated with (+/−)-thioctic acid 250 *μ*mol/kg/day, (E) CCI SHRs treated with (+/−)-thioctic acid 125 *μ*mol/kg/day, (F) CCI SHRs treated with (+)-thioctic acid 125 *μ*mol/kg/day, (G) CCI SHRs treated with (−)-thioctic acid 125 *μ*mol/kg/day, and (H) CCI SHRs treated with pregabalin 300 *μ*mol/kg/day (H). The data of densitometric analysis were expressed as arbitrary units. *P < 0.05 versus WKY control rats; **P < 0.05 versus SHR Sham operated control rats; ***P < 0.05 versus control CCI SHR.

**Figure 4:**
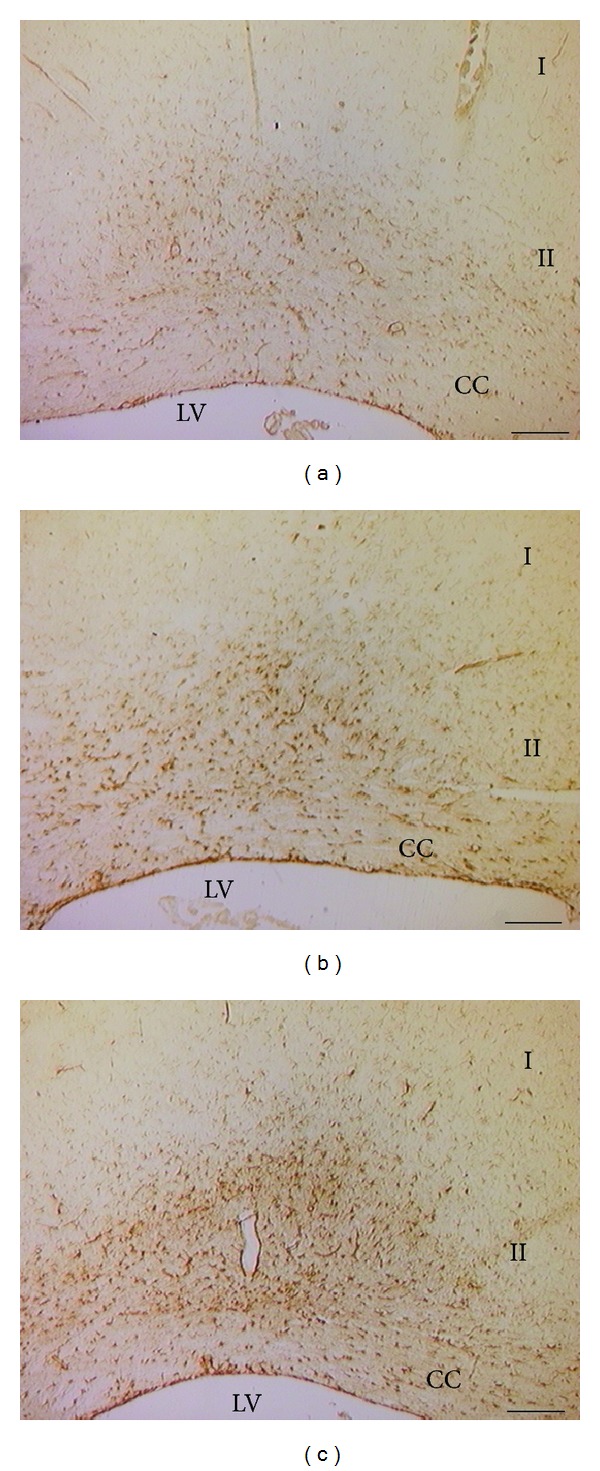
Sections of the sensory cortex (S1HL) processed for the immunohistochemical demonstration of glial fibrillary acidic protein (GFAP). Note the increase of the immunoreactions in the zone II of the cortex in the control Sham-operated SHRs and in larger extent in the control CCI SHRs. (a) WKY control Sham-operated rats, (b) control Sham-operated SHRs, and (c) control CCI SHRs. I: zone 1; II: zone 2; CC: corpus callosum; LV: lateral ventricle. Calibration bar: 200 *μ*m.

**Figure 5:**
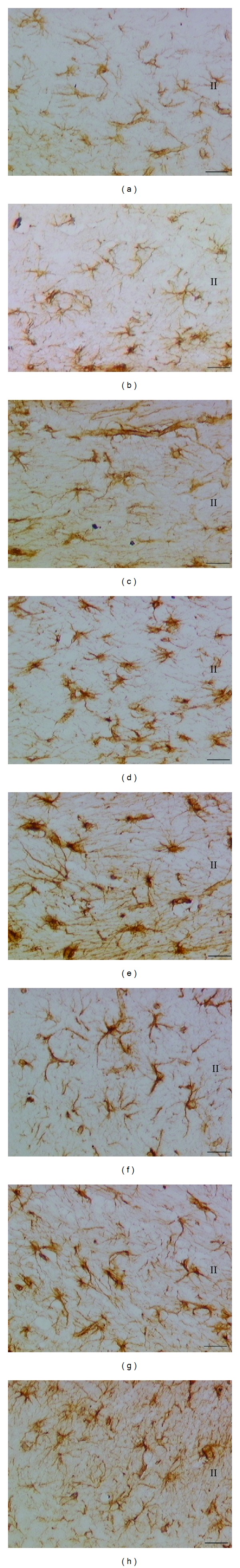
High magnification of the sections of the sensory cortex (S1HL) processed for the immunohistochemical demonstration of glial fibrillary acidic protein (GFAP). (a) Control Sham-operated WKY rats, (b) control Sham-operated SHRs, (c) control CCI SHRs, (d) CCI SHRs treated with (+/−)-thioctic acid 250 *μ*mol/kg/day, (e) CCI SHRs treated with (+/−)-thioctic acid 125 *μ*mol/kg/day, (f) CCI SHRs treated with (+)-thioctic acid 125 *μ*mol/kg/day, (g) CCI SHRs treated with (−)-thioctic acid 125 *μ*mol/kg/day, and (h) CCI SHRs treated with pregabalin 300 *μ*mol/kg/day (H). II: zone 2 of sensory cortex. Calibration bar: 25 *μ*m.

The results of image analysis of the size of GFAP reactive astrocytes are shown in Figures 3–5. In control SHR a significant increase in the size of GFAP immunoreactive astrocytes was observed (Figure 3). This phenomenon was more pronounced in the gray matter of sensory cortex (Figure 3(b)). In WKY rats astrocytes were apparently normal and only few hypertrophic elements were observed (Figures 4(a) and 5(a)). In SHR the presence of hypertrophic elements characterized by hyperreactive astrocytes (H/R) and hypertrophic/hyperimmunoreactive astrocytes (H/H) was observed (Figures 4(b) and 5(b)). After sciatic nerve ligation an increase of GFAP immunoreactive astrocytes was found in the gray and white matter of motor (Figure 3) and sensory cortex (Figures 3, 4(c), and 5(c)). In sensory cortex clusters of H/R and H/H elements were observed in zone 2 near the corpus callosum, where astrocytes were more numerous and characterized by more length cellular processes compared to those of WKY rats (Figures 4(c) and 5(c)).

Treatment with (+/−)-thioctic acid (250 *μ*mol/kg/day) (Figures 3 and 5(d)) and to a greater extent with (+)-thioctic acid (125 *μ*mol/kg/day) (Figures 3 and 5(f)) countered the increase in the volume of GFAP-immunoreactive astrocytes. In sensory cortex (−)-thioctic acid (125 *μ*mol/kg/day) and pregabalin did not affect astroglial reaction (Figures 3, 5(g), and 5(f)).

**Figure 6:**
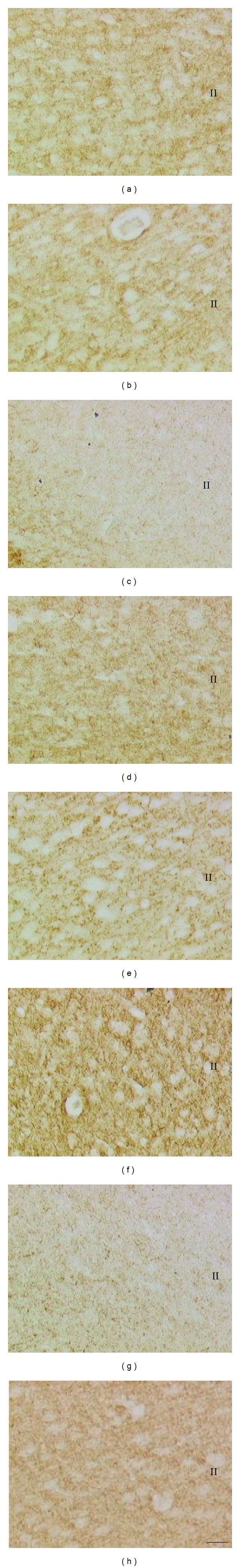
Sections of the sensory cortex (zone 2) processed for the immunohistochemical demonstration of neurofilament 200 kDa. (a) Control Sham-operated WKY rats, (b) control Sham-operated SHRs, (c) control CCI SHRs, (d) CCI SHRs treated with (+/−)-thioctic acid 250 *μ*mol/kg/day, (e) CCI SHRs treated with (+/−)-thioctic acid 125 *μ*mol/kg/day, (f) CCI SHRs treated with (+)-thioctic acid 125 *μ*mol/kg/day, (g) CCI SHRs treated with (−)-thioctic acid 125 *μ*mol/kg/day, and (H) CCI SHRs treated with pregabalin 300 *μ*mol/kg/day (h). II: zone 2 of sensory cortex. Calibration bar: 25 *μ*m.

**Figure 7:**
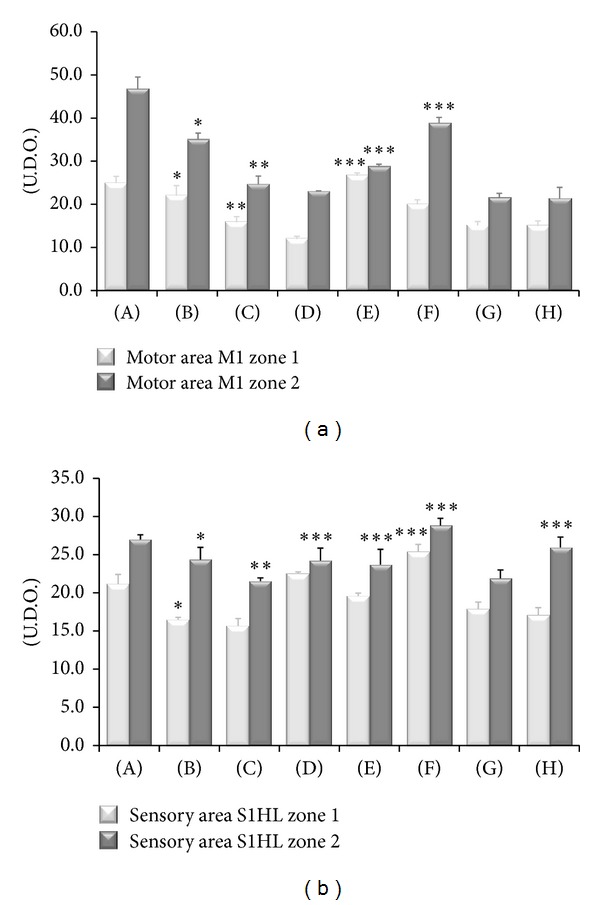
Neurofilament 200 kDa immunoreactivity in the motor and sensory areas of cerebral cortex of the different animal groups investigated. (A) Control Sham-operated WKY rats, (B) control Sham-operated SHRs, (C) control CCI SHRs, (D) CCI SHRs treated with (+/−)-thioctic acid 250 *μ*mol/kg/day, (E) CCI SHRs treated with (+/−)-thioctic acid 125 *μ*mol/kg/day, (F) CCI SHRs treated with (+)-thioctic acid 125 *μ*mol/kg/day, (G) CCI SHRs treated with (−)-thioctic acid 125 *μ*mol/kg/day, and (H) CCI SHRs treated with pregabalin 300 *μ*mol/kg/day (H). Values are expressed in arbitrary units calculated microdensitometrically as detailed in Section 2. Data are the mean ± SE. *P < 0.05 versus WKY control rats; **P < 0.05 versus SHR Sham-operated control rats; ***P < 0.05 versus control CCI SHR.

NFP immunoreactivity was localized in nerve fibre-like structures within motor and sensory cortices (Figure 6). Quantitative image analysis performed in zones 1 (layers I–IV) and 2 (layers V–VI) revealed both in motor and sensory cortex a decrease of NFP-immunoreactive structures in SHR compared to WKY rats (Figure 7). In CCI-operated SHRs a further decrease of NFP immunoreaction was observed (Figures 6(c) and 7). This loss was countered primarily in zone 2 of motor cortex by treatment with (+/−)-thioctic acid (125 *μ*mol/kg/day) and (+)-thioctic acid (125 *μ*mol/kg/day) but not by (−)-thioctic acid (125 *μ*mol/kg/day) (Figures 6 and 7).

In zone 2 of sensory cortex treatment with (+/−)-thioctic acid (250 *μ*mol/kg/day or 125 *μ*mol/kg/day) and (+)-thioctic acid (125 *μ*mol/kg/day) countered the NFP-immunoreaction decrease. Pregabalin increased NFP immunoreactivity in zone 2 of sensory cortex (Figures 6(h) and 7). (+)-Thioctic acid (125 *μ*mol/kg/day) was the only treatment increasing NFP immunoreaction in zone 1 of sensory cortex (Figure 6).

**Figure 8:**
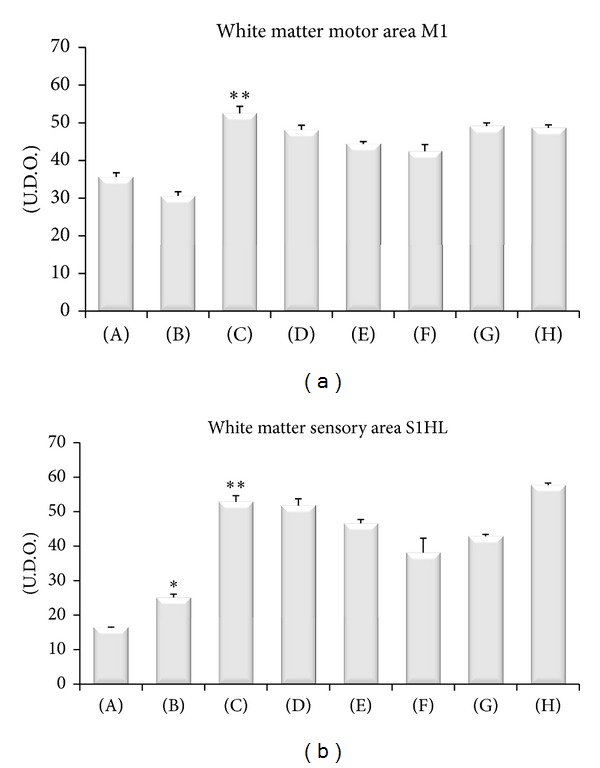
Myelin basic protein immunoreactivity in the white matter of motor and sensory areas of the different animal groups investigated. (A) Control Sham-operated WKY rats, (B) control Sham-operated SHRs, (C) control CCI SHRs, (D) CCI SHRs treated with (+/−)-thioctic acid 250 *μ*mol/kg/day, (E) CCI SHRs treated with (+/−)-thioctic acid 125 *μ*mol/kg/day, (F) CCI SHRs treated with (+)-thioctic acid 125 *μ*mol/kg/day, (G) CCI SHRs treated with (−)-thioctic acid 125 *μ*mol/kg/day, and (H) CCI SHRs treated with pregabalin 300 *μ*mol/kg/day (H). Values are expressed in arbitrary units calculated microdensitometrically as detailed in Section 2. Data are the mean ± SE. *P < 0.05 versus WKY control rats; **P < 0.05 versus SHR Sham-operated control rats.

**Figure 9:**
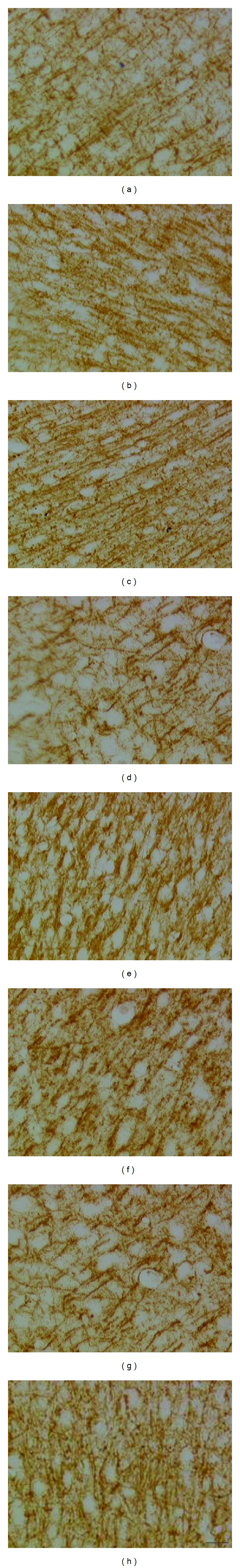
Sections of motor area (M1) processed for the immunohistochemical demonstration of myelin basic protein. (a) WKY control Sham-operated rats, (b) control Sham-operated SHRs, (c) control CCI SHRs, (d) CCI SHRs treated with (+/−)-thioctic acid 250 *μ*mol/kg/day, (e) CCI SHRs treated with (+/−)-thioctic acid 125 *μ*mol/kg/day, (f) CCI SHRs treated with (+)-thioctic acid 125 *μ*mol/kg/day, (g) CCI SHRs treated with (−)-thioctic acid 125 *μ*mol/kg/day, and (h) CCI SHRs treated with pregabalin 300 *μ*mol/kg/day (H). Calibration bar: 25 *μ*m.

Sections processed for MBP immunohistochemistry developed dark-brown staining in the myelin around the axons in zone 2 and in the corresponding white matter of different cerebrocortical areas. The immunoreaction was more pronounced in SHR compared to WKY (Figures 8 and 9). A further increase was seen in CCI-operated SHR compared to control Sham-operated SHR (Figures 8 and 9). Pharmacological treatments with thioctic acid or pregabalin did not affect MBP immunoreactivity (Figures 8 and 9).

**Figure 10:**
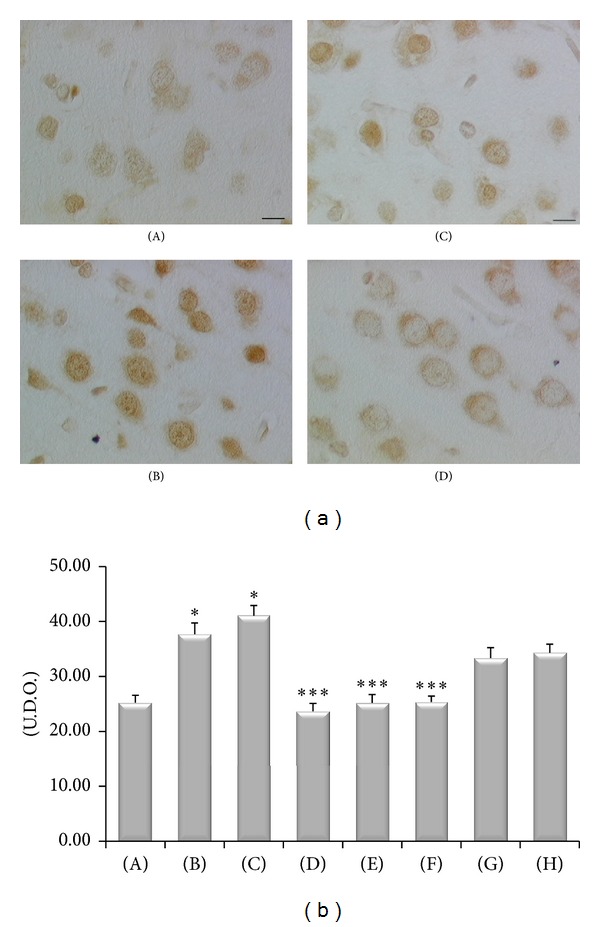
8-Oxo-dG immunoreactivity in the cerebral cortex (layer VI) of the different animal groups investigated (a) and densitometric analysis of the intensity of immunoreaction (histogram). (A) WKY control Sham-operated rats, (B) control Sham-operated SHRs, (C) control CCI SHRs, (D) CCI SHRs treated with (+/−)-thioctic acid 250 *μ*mol/kg/day, (E) CCI SHRs treated with (+/−)-thioctic acid 125 *μ*mol/kg/day, (F) CCI SHRs treated with (+)-thioctic acid 125 *μ*mol/kg/day, (G) CCI SHRs treated with (−)-thioctic acid 125 *μ*mol/kg/day, and (H) CCI SHRs treated with pregabalin 300 *μ*mol/kg/day (H). *P < 0.05 versus WKY control rats; ***P < 0.05 versus control CCI SHR. Calibration bar: 10 *μ*m.

8-Oxo-dG immunostaining was expressed in a thin granular staining localized in the nuclei of cortical neurons. Immunoreaction was more pronounced in SHR compared to WKY and a certain increase of it was observed in CCI-operated SHR compared to control Sham-operated SHR (Figure 10). Treatment with the different doses of (+/−)-thioctic acid and enantiomers tested decreased the 8-oxo-dG immunoreactions (Figure 10).

## 4. Discussion

Peripheral neuropathies are syndromes characterized by nerve damage and degeneration. Their compressive nature is documented in several condition. An example is given by radiculopathy or sciatica which involves lower extremities and is related to disc herniation [27].

Oxidative stress is induced by an imbalance in the cellular redox state, depending either on overproduction of ROS or on dysfunction of the antioxidant systems. Oxidative stress plays an important role in experimental animal models of neuropathic pain. In rats with neuropathy subsequent to spinal nerve ligation, the production of superoxide is increased in dorsal horn neurons [9] and ROS scavengers alleviate neuropathic pain in a reversible manner [12]. Increasing evidence supports the notion that oxidative stress is the biochemical trigger for sciatic nerve dysfunction in rats treated chronically with alcohol [28]. Recent studies indicate that ROS are also involved in the development of persistent pain. The removal of excessive ROS by free radical scavengers, such as phenyl N-tert butylnitrone (PBN) and 4-hydroxy-2,2,6,6-tetramethylpiperidine 1-oxyl (TEMPOL), produced significant analgesic effects both in neuropathic [12, 29] and in inflammatory pain [30]. Furthermore, increased production of ROS [9] and enhanced antioxidant activity [31] were observed in the spinal cord after peripheral nerve injury.

Apart from oxidative stress, nitrosative stress too may play an important role in the pathogenesis of neuropathic pain. Increased levels of nitric oxide synthase (NOS) and nitric oxide (NO) were reported in spinal cord after tissue inflammation caused by capsaicin [32]. This increase is the factor probably maintaining secondary hyperalgesia after capsaicin treatment [32]. Superoxide anions can react with nitric oxide, forming peroxynitrite, which rapidly causes protein nitrosylation, lipid peroxidation, DNA damage, and cell death and has direct toxic effects on nerve tissue [33, 34].

Oxidative stress and endothelial dysfunction are commonly observed in hypertensive individuals [35]. Increasing evidence suggests that they also have a causal role in the molecular processes leading to hypertension. ROS may directly alter vascular function or cause changes in vascular tone by several mechanisms including altered NO bioavailability or signaling [35]. Enhanced oxidative stress and brain vascular injury are documented in SHR [36]. Ligation of sciatic nerve further increases oxidative stress [9] and therefore CCI-operated SHR could represent an elevated oxidative stress model suitable for assessing the potential neuroprotective activity of antioxidants. In this study we have shown that nerve ligation increases oxidative stress, documented by increased plasma levels of MDA, increased oxidation state of the plasma proteins, and decreased SOD activity. SHRs are more sensitive than their normotensive cohort to the increase in oxidative stress induced by nerve ligation, probably due to the impaired oxidative balance induced by arterial hypertension [37]. On the other hand, the experimental paradigm used in this study represents a good model of entrapment (compressive) neuropathy as CCI leads to massive nerve degeneration, with changes of both axonal and myelin components [2, 38, 39].

Thioctic acid was chosen as antioxidant in view of the increasing evidence of its neuroprotective activity in nervous system disorders characterized by vascular injury [17, 40]. The antioxidant activity of the compound is assigned to the (+)-enantiomer [17], although there is not a general agreement on it [41]. The goal of these experiments was to assess the effects of the CCI and of antioxidant or pregabalin treatment on astroglial reaction, MBP, and neurofilament expression in primary motor and sensory cortex of cerebral cortex. The findings that different thioctic acid formulations did not affect blood pressure values in SHR indicate that any activity observed in the brain of SHR is not related to changes in blood pressure.

Our results documented an antioxidant activity of thioctic acid with decreased plasma levels of MDA and a reduction of oxidation of proteins. These findings are consistent with clinical studies reporting a reduction of oxidative stress both in healthy subjects and in patients with diabetic peripheral neuropathy [42]. In diabetic individuals administration of 600 mg/day of thioctic acid for 3 months reduced significantly the formation of products of lipid peroxidation [42].

Monolateral CCI of sciatic nerve increased GFAP expression mainly in the gray matter of sensory cortex and decreased NFP expression as a consequence of nerve damage. Astrocytes play an active role in maintaining the structure, metabolism, and function of the brain [43] and become hypertrophic in response to diverse brain injury. Depending on their activation status, they are also referred to as reactive and/or activated astrocytes [44–46].

Chronic neuropathic pain is accompanied by reorganization and functional changes in CNS cortical and subcortical structures, including the medial prefrontal cortex [16, 47, 48], thalamus [49], amygdala [50], and anterior cingulate cortex [51]. Our study has shown that treatment with antioxidants, but not with pregabalin, prevented to some extent astrogliosis and neuronal damage in cerebral cortex. Comparatively the (+)-thioctic acid enantiomer resulted more effective than (+/−)-thioctic acid, even when the last one is used at concentrations double of those of (+)-thioctic acid. (−)-Thioctic acid was ineffective on brain damage induced by CCI of the sciatic nerve. Effects of (+)-thioctic acid are probably not limited to what we did observe in this study, as the compound contributed to the regeneration of the nerve axonal components and increased the threshold of the nociceptive response to a mechanical stimuli on ligated nerve [39].

The neuroprotective activity of thioctic acid is probably related to its antioxidant activity, as treatment with the compound decreased of 8-oxo-dG immunoreactions in the nuclei of cortical neurons. These findings are consistent with the demonstration of neuroprotective properties of thioctic acid in animal models of brain injury [52, 53].

The most pronounced activity of (+)-thioctic acid is probably related to its more favorable kinetic profile and better plasma bioavailability. Oral administration of (+)-thioctic acid to healthy volunteers results in a kinetic profile similar to that of intravenously administered (+/−)-thioctic acid, although plasma accumulation was quantitatively different for intravenous compared to the oral formulation of the compound [54, 55]. The intravenous is the only administration route of thioctic acid for which controlled studies have clearly documented a clinical efficacy in the treatment of diabetic neuropathy [56]. The antioxidant activity of (+)-thioctic acid is also loftier than the racemic mixture of the compound in cellular models of increased oxidative stress [20]. In PC12 cells exposed to H_2_O_2_ for inducing oxidative stress, treatment with (+)-thioctic acid prevented cell death and promoted growth, whereas the (−)-thioctic acid enantiomer was ineffective and the racemic form was active only at higher doses [20, 57].

## 5. Conclusions

In summary, the demonstration of the activity of thioctic acid, in countering oxidative stress and in protecting CNS from damage induced by a lesion of peripheral nervous system mimicking entrapment neuropathy, suggests that antioxidant strategies may represent a therapeutic approach in the treatment of compressive neuropathies. The greater activity of (+)-thioctic acid and its higher bioavailability after oral administration [54, 55] may represent a stimulus for assessing clinical activity of the compound in controlled studies.

## Figures and Tables

**Table 1:** Systolic blood pressure values.

	Treatment	Before Treatment		After Treatment	
		WKY	SHR	WKY	SHR
Sham	Vehicle	156 ± 7	209 ± 6*	144 ± 9	213 ± 3*
CCI	Vehicle	145 ± 6	220 ± 8*	149 ± 13	232 ± 16*
CCI	(+/−)-Thioctic acid 250 *μ*mol/Kg/day	157 ± 9	198 ± 8*	155 ± 8	207 ± 5*
CCI	(+/−)-Thioctic acid 125 *μ*mol/Kg/day	149 ± 11	208 ± 12*	150 ± 14	205 ± 9*
CCI	(+)-Thioctic acid 125 *μ*mol/Kg/day	148 ± 6	211 ± 11*	136 ± 13	225 ± 8*
CCI	(−)-Thioctic acid 125 *μ*mol/Kg/day	154 ± 4	201 ± 14*	156 ± 9	217 ± 9*
CCI	Pregabalin 300 *μ*mol/Kg/day	150 ± 6	215 ± 5*	146 ± 5	201 ± 9*

The data are expressed as mmHg and are the mean of 3 different measurements. *P < 0.01 versus WKY rats of the same group of treatment.
