# Correction to “Neuroprotective Activity of Thioctic Acid in Central Nervous System Lesions Consequent to Peripheral Nerve Injury”

**DOI:** 10.1155/bmri/9850163

**Published:** 2026-04-08

D. Tomassoni, F. Amenta, L. Di Cesare Mannelli, C. Ghelardini, I. E. Nwankwo, A. Pacini, S. K. Tayebati, “Neuroprotective Activity of Thioctic Acid in Central Nervous System Lesions Consequent to Peripheral Nerve Injury,” *BioMed Research International*, 2013: 985093, https://doi.org/10.1155/2013/985093.

In the article, there is an error in Figure 9, raised directly and on PubPeer [1]. Specifically, there are repeated regions between Panels d and g in Figure 9. The correct Figure 9 is shown below:

Figure 9Sections of motor area (M1) processed for the immunohistochemical demonstration of myelin basic protein. (a) WKY control sham‐operated rats, (b) control sham‐operated SHRs, (c) control CCI SHRs, (d) CCI SHRs treated with (+/−)‐thioctic acid 250 *μ*mol/kg/day, (e) CCI SHRs treated with (+/−)‐thioctic acid 125 *μ*mol/kg/day, (f) CCI SHRs treated with (+)‐thioctic acid 125 *μ*mol/kg/day, (g) CCI SHRs treated with (−)‐thioctic acid 125 *μ*mol/kg/day, and (h) CCI SHRs treated with pregabalin 300 *μ*mol/kg/day (H). Calibration bar: 25 *μ*m.

**Figure:**
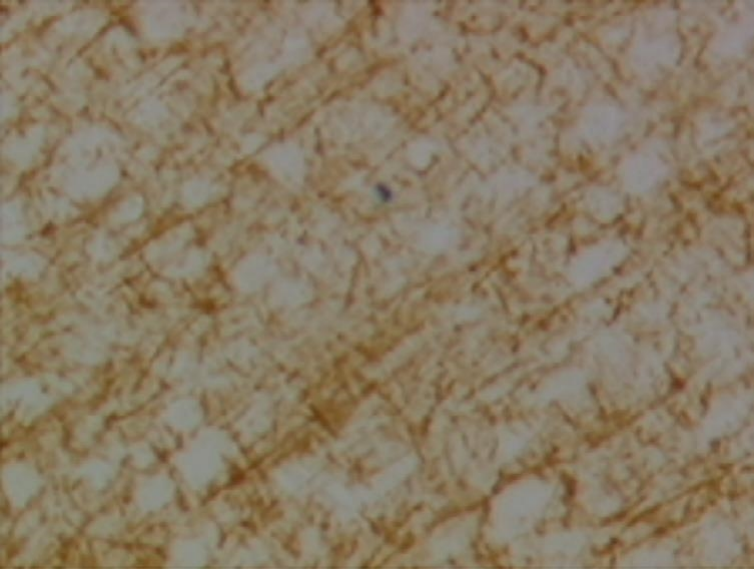
(a)

**Figure:**
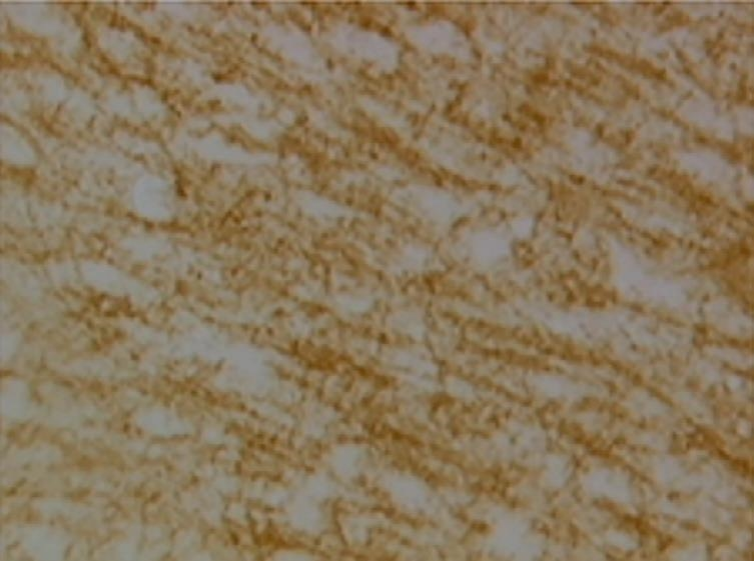
(b)

**Figure:**
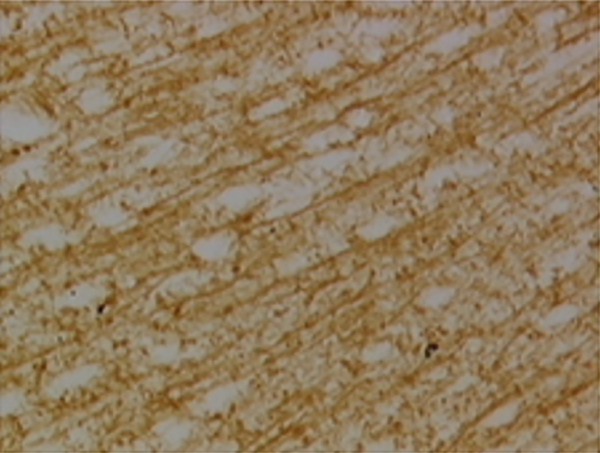
(c)

**Figure:**
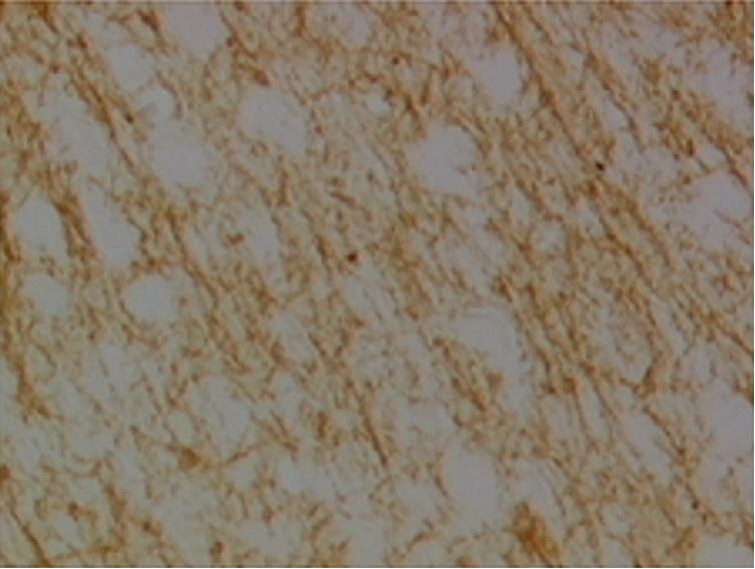
(d)

**Figure:**
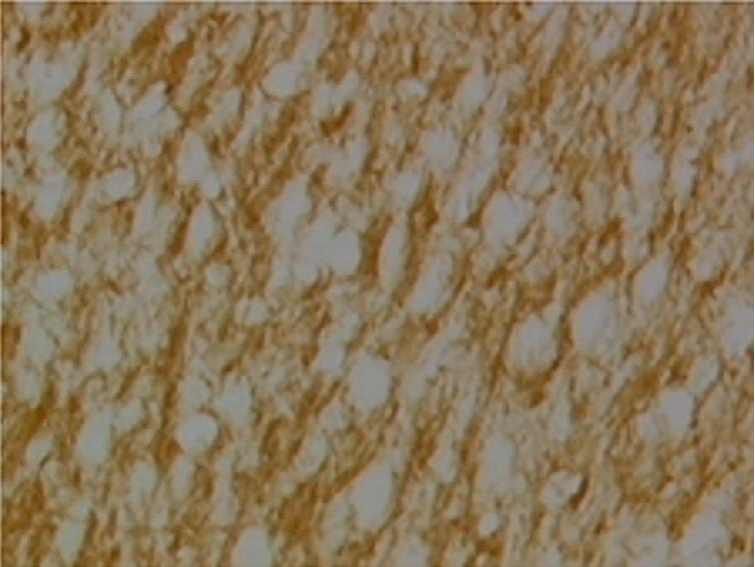
(e)

**Figure:**
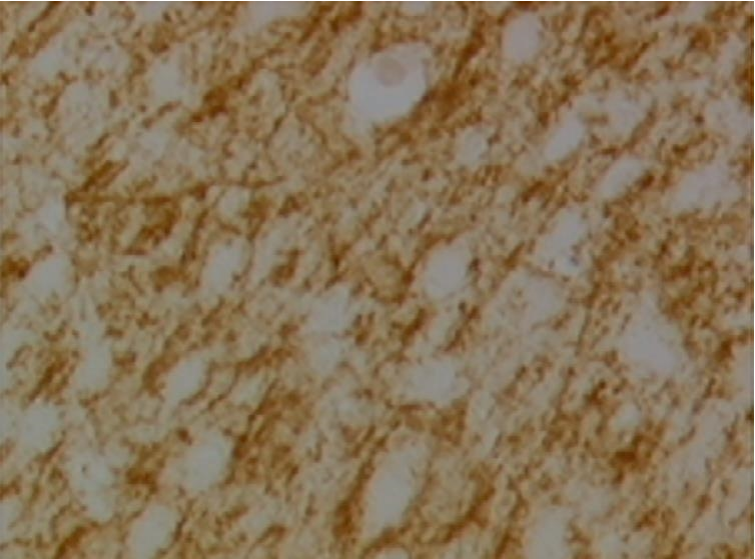
(f)

**Figure:**
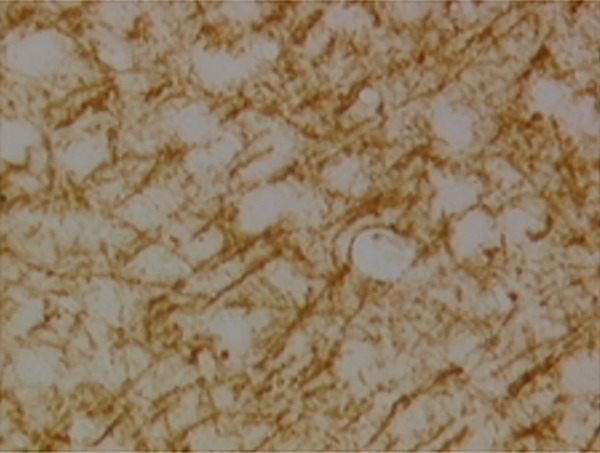
(g)

**Figure:**
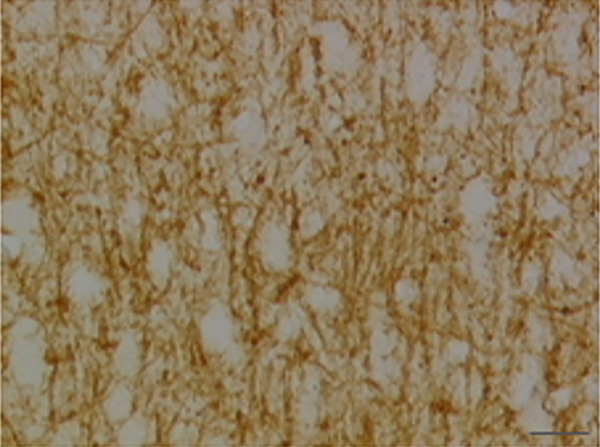
(h)

We apologize for this error.
